# Outbreak of *Mycobacterium orygis* in a Shipment of Cynomolgus Macaques Imported from Southeast Asia — United States, February–May 2023

**DOI:** 10.15585/mmwr.mm7307a2

**Published:** 2024-02-22

**Authors:** Samantha D. Swisher, Sara J. Taetzsch, Mark E. Laughlin, William L. Walker, Adam J. Langer, Tyler C. Thacker, Jessica L. Rinsky, Kimberly A. Lehman, Anne Taffe, Nancy Burton, Doris M. Bravo, Emily McDonald, Clive M. Brown, Emily G. Pieracci

**Affiliations:** ^1^Division of Global Migration Health, National Center for Emerging and Zoonotic Infectious Diseases, CDC; ^2^Epidemic Intelligence Service, CDC; ^3^Division of Tuberculosis Elimination, National Center for HIV, Viral Hepatitis, STD, and TB Prevention, CDC; ^4^Mycobacteria and Brucella Section, National Veterinary Services Laboratories, Animal and Plant Health Inspection Service, U.S. Department of Agriculture, Ames, Iowa; ^5^Division of Field Studies and Engineering, National Institute for Occupational Safety and Health, CDC.

**Summary**

**What is already known about this topic?**

Because nonhuman primates (NHP) can become infected with the same species of *Mycobacteria* that cause human tuberculosis, all NHP imported into the United States are quarantined and screened for tuberculosis. During 2013–2020, no confirmed cases of tuberculosis were diagnosed among NHP during CDC-mandated quarantine.

**What is added by this report?**

During February–May 2023,* Mycobacterium orygis* was detected during CDC quarantine among 26 cynomolgus macaques from a shipment of 540 imported from Southeast Asia. No associated human cases were identified.

**What are the implications for public health?**

Although the zoonotic disease risk to the general population remains low, this outbreak demonstrated the importance of regulatory oversight of NHP importation to prevent the introduction of infectious diseases and protect the health of facility staff members.

## Abstract

Nonhuman primates (NHP) can become infected with the same species of *Mycobacteria* that cause human tuberculosis. All NHP imported into the United States are quarantined and screened for tuberculosis; no confirmed cases of tuberculosis were diagnosed among NHP during CDC-mandated quarantine during 2013–2020. In February 2023, an outbreak of tuberculosis caused by *Mycobacterium orygis* was detected in a group of 540 cynomolgus macaques (*Macaca fascicularis*) imported to the United States from Southeast Asia for research purposes. Although the initial exposure to *M. orygis* is believed to have occurred before the macaques arrived in the United States, infected macaques were first detected during CDC-mandated quarantine. CDC collaborated with the importer and U.S. Department of Agriculture’s National Veterinary Services Laboratories in the investigation and public health response. A total of 26 macaques received positive test results for *M. orygis* by culture, but rigorous occupational safety protocols implemented during transport and at the quarantine facility prevented cases among caretakers in the United States. Although the zoonotic disease risk to the general population remains low, this outbreak underscores the importance of CDC’s regulatory oversight of NHP importation and adherence to established biosafety protocols to protect the health of the United States research animal population and the persons who interact with them.

## Introduction

CDC regulates nonhuman primate (NHP) importation and quarantine under the Public Health Service Act (42 U.S. Code 264). All NHP entering the United States must be imported by CDC-registered facilities and are required to undergo quarantine and tuberculosis testing under 42 Code of Federal Regulations Section 71.53. Imported NHP must have at least three negative tuberculin skin tests (TSTs) using mammalian old tuberculin injected intradermally into the eyelid at a minimum of 2-week intervals before being released from CDC-mandated quarantine. Importers are required to submit samples from any NHP that dies or is euthanized during quarantine and is suspected to have tuberculosis for confirmatory culture. During 2013–2020, 19 imported NHP (0.009% of all NHP imported during this period) received a positive tuberculin skin test result; none had culture-confirmed tuberculosis during the quarantine period.

Sources of NHP imported for research changed markedly during the COVID-19 pandemic: whereas 60% of NHP imported in 2019 came from China, by 2021, imports from China had ceased completely, and 65% of imported NHP originated in Southeast Asia.*

In January 2023, a shipment of 540 captive-bred cynomolgus macaques (*Macaca fascicularis*) was imported by air to the United States from Southeast Asia for research purposes. The macaques were quarantined at a CDC-approved facility, where they were housed in multiple quarantine rooms, each of which had a separate air handling system that maintained manometer-verified negative air pressure relative to the anteroom or hallway. Disinfection protocols for transport and quarantine included the use of a U.S. Environmental Protection Agency-registered tuberculocidal product. Regulations require that transporters and quarantine facility staff members who come within 5 feet (1.52 m) of NHP wear specific personal protective equipment, including a fit-tested, National Institute for Occupational Safety and Health–Approved N95 filtering facepiece respirator or higher-level respirator (42 U.S. Code 71.73). CDC-registered importers must have an occupational health program that includes medical clearance, respirator fit testing and training, and tuberculosis screening at least annually. This report describes the investigation of and response to a positive TST reaction in a macaque, identified on February 7, 2023.

## Investigation and Results

### Identification of *Mycobacterium orygis*

On February 7, 2023, the importer notified CDC of a macaque with a positive TST reaction. The macaque was humanely euthanized, and the carcass underwent postmortem examination, including sample collection for histopathologic and microbiologic testing. Histopathology findings, including acid-fast staining, were consistent with mycobacterial infection, and samples were submitted to U.S. Department of Agriculture’s National Veterinary Services Laboratories (NVSL) for mycobacterial polymerase chain reaction (PCR) testing and culture. PCR testing (*1*) of lung tissue and tracheobronchial lymph nodes for *Mycobacterium tuberculosis* complex (MTBC) was positive; however, because this assay has not been validated for NHP, this finding was not considered confirmatory. The case was confirmed when culture and whole-genome sequencing (WGS) conducted at NVSL later identified the presence of *Mycobacterium orygis*, a species of MTBC that is believed to be most frequently found among humans and animals in South Asia (*2*). WGS and analysis were performed according to methods published in 2021 (*3*), using *M. tuberculosis* H37Rv as the reference. This activity was reviewed by CDC, deemed not research, and was conducted consistent with applicable federal law and CDC policy.^†^

### Additional Cases Among Macaques

In accordance with CDC regulations, the remaining macaques were required to undergo extended quarantine and have at least five additional negative TST results at a minimum of 2-week intervals. On February 21, the importer notified CDC of eight additional macaques with positive TST reactions. Based on preliminary postmortem diagnostics, seven animals were considered to have suspected tuberculosis (Box); all seven were later confirmed to be infected with *M. orygis*, based on culture results of affected tissues and WGS.

BOX **Tuberculosis case definitions for nonhuman primates undergoing diagnostic testing after a positive tuberculin skin test reaction or positive or indeterminate interferon-gamma release assay test result — United States, January–August 2023**

Confirmed*

- Positive MTBC culture result

**Suspected^†^**

- Negative or pending culture result, and
  - Acid-fast bacteria on histopathology, or
  - Positive MTBC PCR test result

**Negative**

- No acid-fast bacteria on histopathology, and
- Negative MTBC PCR, and
- Negative MTBC culture

**Abbreviations:** CFR = code of federal regulations; MTBC = *Mycobacterium tuberculosis* complex; NHP = nonhuman primate; PCR = polymerase chain reaction.

* NHP import regulation (42 CFR 71.53). https://www.ecfr.gov/current/title-42/chapter-I/subchapter-F/part-71/subpart-F/section-71.53.

^†^ Includes diagnostic tests that are considered confirmatory in humans but are not specifically mentioned in the import regulation.

Macaques with positive TSTs were reported until May 30; a total of 32 macaques received a positive TST result during the outbreak (Table). All animals with a positive TST result were humanely euthanized and had samples submitted for postmortem testing, including histopathology with acid-fast staining, MTBC PCR, culture and, when applicable, WGS. Histopathology was performed by a commercial pathology laboratory, and the remaining tests were performed at NVSL. A total of 26 macaques (4.8% of the shipment) received a positive MTBC culture during the outbreak, including 24 (75%) of 32 with a positive TST result and two of 508 (0.4%) with a negative TST result. All isolates were confirmed to be *M. orygis* and shared a common ancestor, which had acquired 85 single nucleotide polymorphisms since the most common ancestor in the NVSL database. Starting early in the outbreak, the importer submitted blood samples from TST-positive macaques to a private laboratory for experimental interferon-gamma release assay (IGRA) testing. In early April, all remaining macaques in the cohort were tested with IGRA, which revealed one positive and four indeterminate results. Two macaques (one positive and one indeterminate) were confirmed by postmortem examination to be culture-positive for *M. orygis*. Overall, macaques receiving positive culture results were identified from 67% of quarantine rooms. The remaining macaques in the cohort were released from quarantine on August 8, 2023, all having received the required five negative TST results after the last TST-positive macaque was removed from the cohort.

**TABLE:** Tuberculosis testing results* among cynomolgus macaques imported from Southeast Asia (N = 540) — United States, January–August 2023

Skin test result	No. of animals (%)^†^
**Positive tuberculin skin test result (n = 32; 6%)**	
Positive IGRA	22 (69)
Acid-fast bacteria on histopathology	23 (72)
Positive MTBC PCR	20 (63)
Positive MTBC culture	24 (75)
**Negative tuberculin skin test result (n = 508; 94%)**	
Positive/Indeterminate IGRA	5 (1.0)
Acid-fast bacteria on histopathology	1 (0.2)
Positive MTBC PCR	2 (0.4)
Positive MTBC culture	2 (0.4)

**Abbreviations:** IGRA = interferon-gamma release assay; MTBC = *Mycobacterium tuberculosis* complex; PCR = polymerase chain reaction.

* If multiple tissues were tested, the animal was considered to have received a positive test result if at least one tissue tested positive.

^†^ Percentages of positive tuberculin skin test results calculated among 32 animals with positive tuberculin skin test results; percentages of negative skin test results calculated among 508 animals with negative tuberculin skin test results.

## Public Health Response

After the importer reported multiple positive TST results and confirmed evidence of active tuberculosis in the first animal with a positive TST, CDC informed the state health department and initiated a human exposure risk assessment. Because of administrative and engineering controls and personal protective equipment required during transport and quarantine, risk for exposure among transporter and quarantine facility staff members was presumed to be low. However, in accordance with CDC regulations, all staff members who worked in affected rooms were required to undergo tuberculosis screening at more frequent intervals after the identification of culture-positive NHP. As of February 2024 (8 months after the last positive macaque was detected), none of these persons has received a positive TST or IGRA test result.

CDC notified the airline and airport staff members and transporters that NHP in the shipment had tested positive for tuberculosis and recommended they contact their health care providers for a risk assessment to determine whether follow-up monitoring was recommended. CDC also recommended that the importer notify recipients of NHP after their release from quarantine about the potential risk for tuberculosis exposure, because tuberculosis can have a long incubation period, and TSTs are subject to false-negative results. A veterinary health alert was distributed in July 2023 to relevant professional organizations, including the Association of Primate Veterinarians, National Association of Animal Health Officials, the National Association of State Public Health Veterinarians, and all CDC-registered NHP importers. CDC also developed a fact sheet about tuberculosis among NHP (CDC, unpublished data, 2023) and distributed it with the veterinary health alert. To date, human infection has not been reported in the United States in connection with this outbreak. No information was available about the tuberculosis status of workers at the supplier facility in Southeast Asia.

## Discussion

Preventing outbreaks of tuberculosis in NHP facilities is important to protect the health of workers and animals and to avoid affecting research outcomes. NHP research facilities in the United States typically have routine tuberculosis screening programs for their employees to reduce the risk for tuberculosis spread to and from their facilities. However, in many cases, it might not be possible to confirm that foreign suppliers follow similar protocols. Because macaques from multiple quarantine rooms were affected, and the isolates were genetically close by phylogenetic analysis, the group is presumed to have been infected from a common human or animal source before importation. This presumption is supported by the fact that the isolate was identified as *M. orygis*, a species of MTBC that has been detected primarily in South Asia (*2*).

*M. orygis* was identified as a separate species of MTBC in 2012 (*4*), and its epidemiology remains poorly understood. *M. orygis* infection has been reported both in humans (*5*) and animals; animal cases have mostly been in ungulates (*6*,*7*), but there has been one report in two captive, wild-caught NHP housed at a zoo (*6*). Most reported cases have had a known connection to South Asia. The prevalence of *M. orygis* in this region is unknown but is suspected to be higher than was previously recognized because common diagnostic tests might not differentiate it from other MTBC species (*2*).

This outbreak highlights the importance of public health oversight for imported NHP. Although current surveillance methods successfully detected this outbreak, tuberculosis screening among NHP can be challenging. The clinical presentation of tuberculosis among NHP varies widely, from asymptomatic infection to acute fulminant and chronic disease (*8*). TST using mammalian old tuberculin is an important component of current import surveillance, but it is an imperfect test, subject to both false-positive and false-negative results (*8*). A testing protocol that incorporates multiple different tests has been suggested as a potential way to improve sensitivity and specificity (*9*,*10*) but, to date, no such protocol has been validated. Rigorous occupational safety and health programs based on the hierarchy of controls (i.e., elimination, substitution, engineering controls, administrative controls, and personal protective equipment)^§^ will remain critical to protect NHP workers, especially those who have close contact with imported NHP. In this outbreak, meticulous adherence to regulations by the importer and a strong public-private partnership between the importer and CDC might have prevented human cases of tuberculosis.

## Footnotes

*
https://www.cdc.gov/importation/bringing-an-animal-into-the-united-states/monkeys.html

† 45 C.F.R. part 46, 21 C.F.R. part 56; 42 U.S.C. Sect. 241(d); 5 U.S.C. Sect. 552a; 44 U.S.C. Sect. 3501 et seq.

§
https://www.cdc.gov/niosh/topics/hierarchy/default.html
